# Spousal discordance on reports of contraceptive communication, contraceptive use, and ideal family size in rural India: a cross-sectional study

**DOI:** 10.1186/s12905-018-0636-7

**Published:** 2018-09-04

**Authors:** Holly B. Shakya, Anindita Dasgupta, Mohan Ghule, Madhusudana Battala, Niranjan Saggurti, Balaiah Donta, Saritha Nair, Jay Silverman, Anita Raj

**Affiliations:** 1 Division of Infectious Disease and Global Public Health, Department of Medicine, University of California, San Diego School of Medicine, San Diego, CA USA; 2 Center on Gender Equity and Health (GEH), University of California, San Diego, CA USA; 3 Social Intervention Group, School of Social Work, Columbia University, New York, NY USA; 4 National Institute for Research in Reproductive Health (NIRRH), Mumbai, India; 5 Population Council, New Delhi, India; 6 Bill and Melinda Gates Foundation, New Delhi, India; 7 National Institute of Medical Statistics, New Delhi, India

**Keywords:** India, Contraceptive use, Fertility preferences, Spousal concordance, Gender inequality, Gender norms

## Abstract

**Background:**

Persistent low rates of spacing contraceptive use among young wives in rural India have been implicated in ongoing negative maternal, infant and child health outcomes throughout the country. Gender inequity has been found to consistently predict low rates of contraception. An issue around contraceptive reporting however is that when reporting on contraceptive use, spouses in rural India often provide discordant reports. While discordant reports of contraceptive use potentially impede promotion of contraceptive use, little research has investigated the predictors of discordant reporting.

**Methods:**

Using data we collected from 867 couples in rural Maharashtra India as part of a men-focused family planning randomized controlled trial. We categorized couples on discordance of men’s and women’s reports of current contraceptive use, communication with their spouse regarding contraception, and ideal family size, and assessed the levels of discordance for each category. We then ran multinomial regression analyses to determine predictors of discordance categories with a focus on women’s empowerment (household and fertility decision-making, women’s education, and women’s knowledge of contraception).

**Results:**

When individuals reported communicating about contraception and their spouses did not, those individuals were also more likely to report using contraception when their spouses did not. Women’s empowerment was higher in couples in which both couples reported contraception communication or use or in couples in which only wives reported contraception communication or use. There were couple-level characteristics that predicted husbands reporting either contraception use or contraception communication when their wives did not: husband’s education, husband’s familiarity with contraception, and number of children.

**Conclusions:**

Overall there were clear patterns to differential reporting. Associations with women’s empowerment and contraceptive communication and use suggest a strategy of women’s empowerment to improve reproductive health. Discordant women-only reports suggest that even when programs interact with empowered women, the inclusion of husbands is essential. Husband-only discordant reports highlight the characteristics of men who may be more receptive to family planning messages than are their wives. Family planning programs may be most effective when working with couples rather than just with women, and should focus on improving communication between couples, and supporting them in achieving concordance in their reproductive preferences.

**Trial registration:**

Clinical Trials Number: NCT01593943, registered May 4, 2012 at clinicaltrials.gov.

## Background

Currently it is estimated that approximately only 54% of married Indian women between the ages of 15–49 use contraception, mostly through female sterilization [1]. These statistics vary considerably according to urban/rural residence, age of woman, parity, number of sons, level of education, standard of living and religion [1–4]. Persistent low rates of spacing contraceptive use among young wives in rural India [1] have been implicated in ongoing negative maternal, infant and child health outcomes throughout the country [5]. Understanding the factors associated with contraceptive use in rural India is therefore essential to the promotion of improved maternal and child health.

Low rates of contraceptive use in rural India, despite an increase in contraceptive access, are largely driven by social norms promoting early and high fertility as well as son preference [2, 6], factors that are closely related to and maintained by patterns of gender inequality [2, 4, 6, 7]. Gender inequality also contributes to low levels of contraceptive use through lack of female autonomy over female contraceptive decision-making, as males tend to report higher fertility norms and more negative attitudes toward contraceptives, relative to females [2, 6]. Alternately, when women report higher levels of autonomy within their households they are more likely to use contraception [8, 9].

To gain an understanding of the factors associated with contraceptive use researchers have mostly relied on reports from women, rather than male partners, as women’s reports are considered more accurate [10]. However, studies in which both men and women are surveyed show discordance in reported preferences and behavior. Answers can diverge on questions regarding personal preferences, such as ideal family size, in which case discordance might be reflective of discord or disagreement within the spousal unit, or answers can diverge on questions regarding objective events, such as contraceptive use, in which case discordance might be reflective of problems with question wording, response bias, interpretation or some combination. Discordance around use is often attributable to men reporting condom use when women do not [11, 12], or women who surreptitiously use contraceptives such as pills without her husband’s knowledge [13].

While difference in preferences are easily attributable to legitimate differences, it is difficult to tease out the underlying issue of discordance when the survey questions refer to objective events such as communication around contraceptive use, or actual contraceptive use. This is a serious measurement issue, and is also interesting from the perspective of couple dynamics and how they might inform differences in individual reporting. This sort of discordance may stem from two distinct but potentially overlapping issues. First, if couples are not answering questions in a way that is internally consistent then there may be a serious problem with question validity, reliability or both. In other words, the questions are not being asked such that married men and women would interpret and answer the questions in the same way. This could simply be an issue of question wording. For instance, when a couple is asked whether they “currently use contraception”, it may be difficult to know how to answer if they sporadically use condoms or sometimes practice withdrawal, resulting in discordant answers as a result of misunderstanding the intent of the question (6). Ghuman and colleagues (7) found substantial disagreement within couples on questions regarding women’s autonomy, which they partially ascribed to cognitive differences in how men and women understood the question responses. Questions could be re-worded for more accurate interpretation.

The second potential issue is one of social dynamics. Perhaps it is not the wording of the question itself that is at the root of divergent answers, but issues intrinsic to the couple, such as a higher tendency towards social desirability bias in one spouse versus another, differing interpretations of family reality informed by gendered experiences, or information held by one spouse and not the other. For example, women might be clandestinely using birth control such as pills without the knowledge of her husband, which has been known to occur within India (8). Many studies show that the discordance in contraceptive use reporting is more likely to be an issue of men reporting use when women do not, and that differential reporting tends to cluster around men’s reporting of condom use (6, 9–11). In these cases, women may be underreporting condom use, perhaps due to response bias, and men may be over-reporting. An understanding of what predicts discordance, can enable programs to identify couples in which reporting might be socially biased in one way or another, and whether rewording or further instrument testing might improve confidence in the reports that are given.

In order to improve the interpretation of data around contraceptive use and inform policies to promote reproductive health, therefore, having an accurate depiction of the prevalence and determinants of contraceptive use is essential. This accurate depiction, therefore, can only be obtained through an understanding of phenomenon of discordant reporting and the couple-level factors that predict it. By not asking both partners questions regarding contraceptive use, contraceptive communication and fertility preferences, we have less insight into the dynamics supporting contraceptive use and limit our opportunities to promote it with both men and women. Gender specific insights are particularly important given the role of gender inequality in low rates of spacing and its subsequent outcomes.

While a scattering of few previous studies have analyzed these dynamics, much work remains to be done. For instance, while partner communication on family planning is consistently associated with women’s reports of contraceptive use [14–16], research provides little clarity on spousal discordance regarding these reports. One study by Becker and Costenbader show that discordance in contraceptive use reporting is predicted by lower women’s education, and lack of reported spousal communication on contraception use [11]. Previous studies have shown conflicting associations around discordant fertility preferences, with work from Asia suggesting that women’s fertility preferences (versus men’s) are predictive of unmet need for family planning and subsequent unintended pregnancies [17, 18], in contrast to findings from Ethiopia and Kenya indicating that men’s fertility preferences are mostly strongly associated with contraceptive use [14, 19, 20].

Given previous work showing that gender inequality is a significant predictor of contraceptive use behaviors, it is reasonable to hypothesize that gender inequality may also predict contraceptive use, contraceptive communication, and fertility preference discordance. A five country study in Asia, however, found that community level women’s empowerment measures **did not** predict differences in fertility preferences [17]. While not directly related to contraceptive use, work in Nepal and Guatemala, suggests that spousal discordance in reporting wife’s autonomy is related to discordance in reports of family health care utilization [21, 22]. Despite the importance of women’s autonomy on contraceptive use, little research, however, has examined the inter-relationship between female autonomy and discord in reported contraceptive practices among spouses.

This study assesses the predictors of discordance in reports of fertility preferences as well as contraceptive communication and use in a sample of young married couples in rural India with a focus on women’s empowerment indicators as correlates of discordance outcomes.

## Methods

We analyzed baseline data from non-pregnant couples (*N* = 867) participating in the CHARM (Counseling Husbands to Achieve Reproductive Health and Marital Equity Study) intervention, a family planning evaluation study conducted in Maharashtra, India [23].

### Data

#### The CHARM study

The CHARM intervention, was a male-centered family planning intervention for young couples in rural Maharashtra, India. Study participants (*N* = 1081) were assessed via survey at baseline, 9 and 18 months post-baseline. Present analyses used data from the baseline assessment of the wives and are restricted to those who were not pregnant (*n* = 867).

#### Study setting

Study participant recruitment took place in 50 clusters in the Thane District of Maharashtra. Geographic clusters were selected using a process of community mapping based on geographic boundaries (eg. hill, roads, streams), population density (each cluster had to have 300 households and the presence of a private health care provider). The clusters were randomized equally to the CHARM intervention or control conditions (who received referrals to local existing family planning programs) to assess treatment impact on spacing contraceptive use, pregnancy, and unmet family planning need.

#### Study recruitment

Between March and December 2012, trained research staff approached households to identify young married men between 18 and 40 years of age within the selected clusters. If a married couple with a man in the specified age range was home, research staff provided details regarding evaluation study and CHARM intervention participation. If the couple indicated interest in participating, research staff would conduct the informed consent process with the couple in a private space in the house. Due to low literacy rates in the population, consent forms were read to participants in full, and then participants were asked to sign their consent. Participants were told whether or not they would receive the CHARM intervention or control condition at the time of informed consent; hence, researchers were not blinded to treatment conditions. Once the informed consent process was complete, couples were screened for eligibility. Eligibility criteria included being 18–40 years of age, fluency in Marathi, residing with their wife in the cluster area for the past 3 months, plans to stay in the cluster for another 2 years, and no sterilization for either the man or his wife. If a couple was eligible for participation, research staff again described study procedures and asked if the couple was willing to participate in the full CHARM intervention evaluation study, as well as CHARM intervention program for those residing in intervention clusters. Research staff screened 1881 couples between March and December 2012. Of those couples screened, 1143 were eligible to participate in the study (60.8% eligibility rate), 1081 eligible couples chose to participate in the study (94.6% participation rate). Sample size for these analyses was determined by the sample needed to detect an effect in the original trial.

#### Data collection

After couples completed eligibility screening and informed consent procedures, sex-matched research staff administered a 60-min paper survey with husbands and wives separately. Survey items covered a broad range of topics including demographics, contraception knowledge and use, marital communication, sexual history, and gender equity attitudes. Wives were also given a urine pregnancy test at baseline. Subsequent to baseline survey completion, husbands were linked with village health care providers to receive the first session of the CHARM Intervention. No monetary incentive was provided for study or intervention program participation.

### Measures

#### Outcome variables

To measure *fertility preference*, operationalized here as ideal family size, we asked both the husband and the wife: “If you could go back to the time you did not have any children and could choose exactly the number of children to have in your whole life, how many would that be?” Spousal *contraceptive communication was* assessed based on whether, in the past 3 months, husbands and wives discussed the use of contraception with their spouse (“yes” or “no”), and spousal *contraceptive use was based on* whether they currently use contraceptives with their spouse (“yes” or “no”), in the past 3 months.

*Spousal discordance in contraceptive use and spousal discordance in contraceptive communication* were constructed by creating four categories for each variable: “*concordant-no*”, “*concordant-yes*”, “*Husband + discordant*” (husband reports the behavior while the woman does not), and “*Wife + discordant*” (wife reports the behavior while the man does not). *Discordance in ideal family size* was calculated using the difference between a husband’s and wife’s reported ideal family size. Responses were then categorized as “*Wife + discordant*” (wife wanting more children), “*Husband + discordant*” (husband wanting more children), and “*concordant*” (both wanting the same).

#### Independent variables (women’s empowerment indicators)

We used variables of wife’s autonomy, equality in fertility decision making, female education in years, and women’s knowledge of contraceptive use as women’s empowerment indicators. To measure *wife’s autonomy*, we asked women three questions on “who makes decisions regarding:” 1) “household needs”, 2) “major household purchases”, and 3) “the wife’s visits to relatives” [1]. Response options were “husband”, “wife”, “husband/wife together”, or “other.” We assigned each question a 1 if respondents answered either “wife” or “husband/wife together”, and 0 if otherwise. Cronbach’s alpha on the women’s measure was 0.91. We also asked women a single yes/no question on whether or not they believed that they had an “equal right with their spouse to decide the number of children to have”, to assess perceived *equality in fertility decision making*. *Knowledge of contraceptive methods* was assessed by asking women to list the main forms of contraception of which they were aware (surveyors did not read answer choices to the respondent). A score of one was assigned to each method reported, and summed them to create a continuous measure of contraceptive method knowledge [24].

#### Covariates

Sociodemographic variables included continuous measures of *age* for both men and women, men’s education, and a four category measure of *caste*-scheduled caste, scheduled tribe, other backwards caste, or other. Family economic status was assessed via *household food insecurity* and *household room number*, both measures previously validated to predict household standard of living [25, 26]. Household food insecurity was assessed via a binary measure of whether anyone in the household “went to bed hungry” or “went the whole day without eating within the last month”; if either husband or wife reported “yes” on these items, the household was classified as food insecure. We also assessed *female employment* based on women’s response to a single “yes”/“no” item on whether they were engaged in “personal income producing activities”. Women were asked about indicators of a traditional family, including having had their marriage *arranged* by their family and living in an *extended family*; the latter has been linked to lower likelihood of using contraception [27, 28]. Women’s reports on *number of living children* was assessed via items on number of living boys and girls, which we summed. Men’s contraceptive knowledge was calculated as for women (see above).

### Statistical analyses

We assessed level of discordance on spousal reports of contraceptive behaviors and fertility preferences using Cohen’s Kappa (K) for categorical variables and Lin’s concordance correlation coefficient (CCC) for continuous variables. Using the criterion proposed by Landis and Koch [29, 30], we categorized correlation coefficients according to the strength of agreement: Poor < 0.00, Slight 0.00–0.20, Fair 0.21–0.40, Moderate 0.41–0.60, Substantial 0.61–0.80, Almost Perfect 0.81–1.00.

To assess predictors of discordance, we took a two-pronged approach. We first considered the crude differences between concordant and discordant couples using multivariate logistic regression analyses, including all predictor variables in the models. While this gives an idea of general trends in these associations, a more nuanced view would consider how the discordance occurs. Then using multinomial logistic regression we looked more carefully at these differences by comparing positive couples, in which one or more of the spouses reported “yes”, versus *concordant*-*no* couples (referent group). In these models we split positive couples into three separate categories depending upon who gave a positive report: “both Yes”, “Men+”, and “Women +”.

For *ideal family size*, we first modeled couples in which their ideal family size is discordant versus concordant (Model 1) using multivariate logistic regression analysis. In Model 2, we conducted multivariate multinomial regression comparing *Husband + discordant* couples and *Wife + discordant* couples, respectively, to *concordant-yes* couples (referent group). Similarly, two models were constructed for *contraception communication* and *contraceptive use* outcomes. Again, Model 1 for each outcome used multivariate logistic regression to compare discordant and concordant couples, and Model 2 used multivariate multinomial regression to compare *concordant-yes* couples, *Husband + discordant* couples, and *Women + discordant* couples, respectively to *concordant-no* couples (referent group), for each outcome. Multivariate Models 1&2 for the discordant contraceptive use outcome also included *ideal family size discordance* and *contraceptive communication discordance* as correlates.

We tested each model’s *Variance Inflation Factor* (VIF) using the VIF function in the package rms, (R version 3.1.1) in order to ensure that issues of multicollinearity were not contaminating our results. Variables with a VIF over 5 were omitted from the multivariate analyses [31].

## Results

### Summary statistics

Table 1 shows summary statistics of study respondents. Women were on average 22.60 years old (Standard deviation [SD] 2.47) vs 26.20 (SD 2.69) for their husbands. The majority of respondents (68%) were scheduled tribe, with 73% of them living with extended family. Over half of the women (56%) reported feeling that they had fertility rights, 24% of them engaged in income generating activities.

**Table 1:** Summary statistics Charm baseline survey married couples rural Maharashtra *N* = 867, excluding those with pregnant women

	Women *n* = 867	Men *n* = 867	Couple
Age in year mean (SD)	22.60 (2.47)	26.20 (2.69)	
Education in years mean (SD)	6.55 (4.17)	7.37 (3.67)	
Caste Scheduled Caste	4%	7%	
Caste Scheduled Tribe	68%	66%	
Caste OBC	24%	19%	
Caste Other	4%	8%	
Wife engaged in income-generating activities	24%		
Household food insecurity (past month)			11%
House size: # of rooms mean (SD)	2.78 (1.37)		
Wife fertility rights	56%		
Women’s autonomy (agreement on 0–3 questions)			
0	20%		
1	2%		
2	12%		
3	66%		
# of contraceptive methods known (SD)	3.66 (2.32)	3.92 (1.48)	
Living with extended family		73%	
Arranged marriage			83%
# of children			
1			18%
2			9%
3			26%
4			6%
5			1%

### Concordance in contraceptive behaviors and ideal family size

Current contraception use reports showed moderate concordance (see Table 2). In only 17% of couples both husbands and wives indicated that they were currently using contraception, while in 68% both agreed that they were not using any contraception. *Husband + discordance* in use, largely in the form of reported condom use, was more likely to be reported than *Wife + discordance* in use (10% vs. 5%). *Wife + discordance* was equally divided between reports of using the pill and male condom use. Contraceptive communication concordance and fertility preferences concordance were fair. Only 18% of couples dually reported discussing contraception in the past 3 months, while 46% reported no communication. *Wife + discordance* in communication was more likely to be reported than *Husband + discordance* in communication (27% vs. 9%). Fifteen percent of men and 11% of women preferred more children than their spouse.

**Table 2:** Agreement within couples on contraceptive use, communication with spouse on contraception within last 3 months, and ideal family size, *N* = 867 couples excluding those with pregnant women

	Concordant Yes	Concordant No	Women+ Discordant	Men+ Discordant	Kappa	Agreement^1^
Contraceptive Use	17%	68%	5%	10%	0.59 (0.53–0.65)	Moderate
Methods used						
Pills	7%	87%	4%	2%	0.64 (0.55–0.72)	Substantial
IUD	2%	98%	0%	0%	0.91(0.84–1.00)	Almost Perfect
Male condom	10%	76%	3%	11%	0.51 (0.44–0.58)	Moderate
Contraceptive Communication	18%	46%	27%	9%	0.25 (0.19–0.31)	Fair
	Mean Women	Mean Men	Avg Diff		CCC	
Optimal number of children	2.04	2.09	0.06		0.30 (0.24–0.35)	Fair
	(SD 0.42)	(SD 0.53)	(SD 0.57)			
Range	(1,4)	(0,5)	(−2,3)			
	Women+Discordant	Men+ Discordant	Concordant			
Fertility Preferences	11%	15%	74%			

Tables 2 and 3 show the results of our regression models, including adjusted odds ratios and 95% confidence intervals for each association.

**Table 3:** Odds ratios (95% CI) from a multivariate logistic regression analysis and a multinomial logistic regression analysis looking at the predictors of differences in fertility preferences within couples, rural Maharashtra India, *N* = 867 couples

	Multivariate logistic regression	Multinomial multivariate logistic regression	
	Model 1	Model 2	
	Discordant vs concordant	Men+ discordant vs. concordant	Women+ discordant vs. concordant
Women’s empowerment			
Equality in fertility decision-making	1.01 (0.69, 1.48)	0.80 (0.49, 1.30)	1.38 (0.80, 2.38)
Women’s Autonomy (0–3)	0.93 (0.81, 1.09)	0.98 (0.81, 1.19)	0.87 (0.71, 1.07)
Wife’s Educ in years	0.95 (0.90, 1.01)	1.00 (0.93, 1.08)	0.90** (0.83, 0.98)
Wife’s Contraceptive familiarity (0–10)	1.03 (0.94, 1.13)	0.97 (0.86, 1.10)	1.08 (0.96, 1.22)
Covariates			
Husband’s Contraceptive familiarity	0.93 (0.80, 1.07)	0.86 (0.71, 1.04)	1.02 (0.84, 1.24)
Extended Family Yes	1.07 (0.71, 1.62)	0.92 (0.56, 1.53)	1.26 (0.67, 2.38)
Arranged Marriage Yes	0.89 (0.56, 1.42)	0.73 (0.41, 1.29)	1.19 (0.60, 2.37)
Number of Children	1.51*** (1.18, 1.94)	1.86*** (1.36, 2.54)	1.13 (0.79, 1.62)
Wife’s Age in years	0.92* (0.83, 1.01)	0.91 (0.80, 1.04)	0.91 (0.80, 1.05)
Husband’s Age in years	1.07 (0.97, 1.17)	1.07 (0.95, 1.20)	1.06 (0.94, 1.20)
Husband’s Educ in years	0.98 (0.92, 1.04)	0.98 (0.91, 1.06)	0.97 (0.89, 1.06)
House size # of rooms	1.00 (0.87, 1.14)	0.99 (0.82, 1.19)	1.00 (0.83, 1.20)
Wife’s income activity	0.76 (0.48, 1.17)	0.72 (0.41, 1.27)	0.78 (0.41, 1.47)
Food insecurity	1.86** (1.09, 3.15)	2.86*** (1.52, 5.39)	1.02 (0.46, 2.28)
Caste: (ref: Scheduled caste)			
Scheduled Tribe	0.66 (0.33, 1.36)	0.48* (0.20, 1.15)	1.14 (0.38, 3.41)
Other Backward Caste	0.77 (0.34, 1.77)	0.45 (0.15, 1.30)	1.54 (0.46, 5.18)
Other	0.79 (0.32, 1.95)	0.49 (0.16, 1.51)	1.60 (0.41, 6.23)

*p < 0.05; **p < 0.01, ***p < 0.001 all estimates are adjusted for all other variables in the model and include cluster level fixed effects not shown

### Ideal family size (Table 3)

None of the women’s empowerment variables were associated with discordance generally (Model 1). However, it is less likely that a wife prefers more children (*Wife + discordant*) than her husband in couples in which wives have higher education.

### Contraceptive communication (Table 4)

**Fig. 1:**
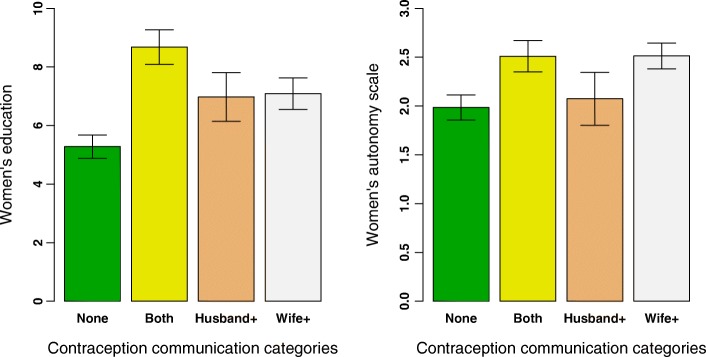
The left panel shows the mean education level reported by women across all four contraceptive communication categories. The right panel shows the mean value of the women’s autonomy scale across all four contraceptive communication categories. In both examples, when both couples report communication or women only report communication, the mean values are higher than for couples in which neither report communication suggesting a positive relationship between women’s empowerment and their communication regarding contraception use. Note also that in couples with more educated women, men are also more likely to report discussing contraception when their wives do not

Several women’s empowerment factors were associated with discordance in contraceptive communication, including women’s autonomy, women’s education, and women’s knowledge of contraceptive methods. In Model 2, we found that for each one point increase in women’s autonomy couples were 1.5 times more likely to both report contraception communication than *both not* report communication (AOR 1.58 95% CI (1.25, 2.00). For each additional year of women’s education couples were 1.2 times more likely to both report contraception than *both not* report communication (AOR 1.18 95% CI (1.08, 1.29). Finally for each point increase in women’s knowledge of contraceptive methods couples were 1.5 times more likely to both report contraception than *both not* report communication (AOR 1.53 95% CI (1.33, 1.75). These factors were also associated with increased likelihood of *Wife + discordant* reporting communication versus both reporting no communication (*concordant-no*). Couples in which women reported equality in contraceptive decision-making were 2.2 times more likely to have women only reporting contraception communication than *both not* report communication (AOR 2.17 95% CI (1.36, 3.48). Figure 1 shows the mean education and mean women’s autonomy scores for couples across all contraception communication categories. Men’s education, men’s contraceptive familiarity, and number of children are also significantly correlated with both *concordant* communication reporting, and *Husband + discordant* reporting.

**Table 4:** Odds ratios (95% CI) from a multivariate logistic regression analysis and a multinomial logistic regression analysis looking at the predictors of differences in reported contraceptive communication within couples, rural Maharashtra India, *N* = 848 couples

	Multivariate logistic regression	Multinomial multivariate logistic regression		
	Model 1	Model 2		
	Discordant vs concordant	Concordant Positive communication vs. Concordant Negative	Discordant Men + communication vs. Concordant Negative	Discordant Women + communication vs. Concordant Negative
Women’s empowerment				
Equality in fertility decision-making	1.31 (0.91, 1.88)	1.53 (0.87, 2.68)	0.66 (0.35, 1.24)	2.17*** (1.36, 3.48)
Wife’s Autonomy	1.11 (0.97, 1.29)	1.58*** (1.25, 2.00)	1.08 (0.85, 1.36)	1.38*** (1.14, 1.68)
Wife’s Educ in years	1.03 (0.98, 1.09)	1.18*** (1.08, 1.29)	1.06 (0.96, 1.17)	1.09** (1.01, 1.17)
Wife’s Contraceptive familiarity	1.05 (0.96, 1.14)	1.53*** (1.33, 1.75)	0.95 (0.79, 1.14)	1.38*** (1.23, 1.56)
Covariates				
Husband’s Contraceptive Knowledge	1.01 (0.89, 1.15)	1.32*** (1.07, 1.62)	1.28** (1.01, 1.63)	1.10 (0.92, 1.33)
Extended Family	1.08 (0.73, 1.60)	1.07 (0.57, 1.99)	0.74 (0.37, 1.47)	1.21 (0.73, 2.01)
Arranged Marriage	1.02 (0.67, 1.56)	0.73 (0.39, 1.38)	0.84 (0.39, 1.83)	0.86 (0.49, 1.51)
Number of Children	1.90*** (1.50, 2.43)	2.41*** (1.66, 3.51)	2.19*** (1.43, 3.34)	2.73*** (2.00, 3.74)
Wife’s Age in years	0.92* (0.84, 1.01)	0.91 (0.79, 1.04)	0.91 (0.77, 1.07)	0.89* (0.79, 1.00)
Husband’s Age in years	0.96 (0.89, 1.05)	1.04 (0.91, 1.18)	0.90 (0.77, 1.04)	1.01 (0.91, 1.13)
Husband’s Educ in years	1.05 (0.99, 1.11)	1.11** (1.01, 1.22)	1.17*** (1.05, 1.29)	1.05 (0.97, 1.13)
House size # of rooms	0.90 (0.79, 1.03)	1.00 (0.82, 1.22)	0.94 (0.75, 1.19)	0.92 (0.77, 1.09)
Wife’s income activity	1.05 (0.69, 1.58)	1.43 (0.75, 2.74)	1.02 (0.47, 2.23)	1.31 (0.76, 2.26)
Food insecurity Yes	0.70 (0.40, 1.22)	0.68 (0.28, 1.67)	0.49 (0.16, 1.46)	0.66 (0.33, 1.33)
Caste: (ref Scheduled caste)				
Scheduled Tribe	1.44 (0.72, 3.01)	3.11* (0.98, 9.90)	2.02 (0.60, 6.78)	2.07 (0.80, 5.33)
OBC	2.10* (0.95, 4.80)	3.41* (0.96, 12.13)	7.09*** (1.85, 27.14)	2.59* (0.88, 7.64)
Other	1.26 (0.53, 3.06)	2.73 (0.70, 10.67)	4.22** (1.01, 17.63)	1.37 (0.42, 4.49)

*p < 0.1; **p < 0.05; ***p < 0.001, all estimates are adjusted for all other variables in the model

### Current contraceptive use (Table 5)

Model 1 indicated that discordance in contraceptive communication and discordance in fertility preferences were associated with discordance in reported contraceptive use. Compared to couples who both reported no communication, couples reporting contraceptive communication in all categories, were more likely to be discordant on reported contraceptive use than concordant (*concordant-yes* 2.8 times more likely; *Wife + discordant* 1.75 times more likely; and *Men +* discordant 9.9 times more likely). Couples in which women preferred a larger ideal family size than their husbands (*Wife + discordant*) compared to couples who preferred the same size family were 1.8 times more likely (AOR 1.88 95% CI [1.00, 3.45]) to be discordant on reported contraceptive use, relative to those who were *concordant* on ideal family size. In terms of women’s empowerment variables, only wife’s education was associated with contraceptive use reporting; couples in which women have more years of education were more likely to be discordant on reported contraceptive use.

**Table 5:** Odds ratios (95% CI) from a multivariate logistic regression analysis and a multinomial logistic regression analysis looking at the predictors of differences in reported current contraceptive use within couples, rural Maharashtra India, *N* = 843 couples

	Multivariate logistic regression	Multinomial multivariate logistic regression		
	Model 1	Model 2		
	Discordant vs concordant	Concordant contraceptive use	Discordant Men+ contraceptive use	Discordant Women+ contraceptive use
Contraceptive communication: (ref concordant no communic)				
Concordant communication	2.86*** (1.48, 5.58)	57.86*** (25.56, 131.02)	10.12*** (4.26, 24.05)	24.87*** (6.82, 90.63)
Discordant Men+ communication	9.90*** (4.97, 20.07)	6.05*** (2.40, 15.25)	18.95*** (8.28, 43.38)	0.76 (0.07, 8.59)
Discordant Women+ communication	1.74* (0.97, 3.14)	8.18*** (4.00, 16.70)	0.95 (0.42, 2.15)	9.29*** (3.23, 26.71)
Fertility Preferences: (ref concordant)				
Discordant Men+ preferences	0.85 (0.44, 1.57)	0.79 (0.38, 1.61)	0.58 (0.24, 1.40)	1.92 (0.68, 5.38)
Discordant Women+ preferences	1.88** (1.00, 3.45)	0.87 (0.38, 1.96)	1.31 (0.56, 3.08)	3.92** (1.32, 11.60)
Women’s empowerment				
Equality in fertility decision-making	1.52 (0.99, 2.34)	2.14** (1.33, 3.44)	1.78* (1.05, 3.01)	2.55* (1.17, 5.54)
Wife’s Autonomy	0.96 (0.81, 1.15)	0.95 (0.78, 1.17)	0.93 (0.75, 1.16)	1.01 (0.74, 1.38)
Wife’s Educ in years	1.10** (1.03, 1.19)	1.04 (0.96, 1.13)	1.14** (1.04, 1.25)	1.08 (0.96, 1.21)
Wife’s Contraceptive familiarity	1.03 (0.93, 1.14)	1.01 (0.90, 1.13)	1.02 (0.89, 1.17)	1.09 (0.93, 1.28)
Covariates				
Contraceptive familiarity H # reported	0.85* (0.71, 1.03)	1.17 (0.95, 1.44)	0.93 (0.72, 1.19)	0.91 (0.64, 1.30)
Extended Family	0.81 (0.47, 1.40)	0.55* (0.30, 1.01)	0.74 (0.36, 1.51)	0.41* (0.15, 1.11)
Arranged Marriage	2.04** (1.13, 3.85)	0.68 (0.37, 1.25)	2.24* (0.94, 5.34)	1.21 (0.44, 3.31)
Number of Children	1.64*** (1.16, 2.31)	1.30 (0.90, 1.88)	2.22*** (1.41, 3.47)	1.30 (0.71, 2.37)
Wife’s Age in years	0.90 (0.79, 1.02)	1.07 (0.94, 1.22)	0.81** (0.68, 0.97)	1.09 (0.88, 1.34)
Husband’s Age in years	0.99 (0.88, 1.11)	1.01 (0.89, 1.14)	1.01 (0.87, 1.18)	0.99 (0.80, 1.21)
Husband’s Educ in years	1.06 (0.98, 1.15)	1.10** (1.01, 1.21)	1.16*** (1.04, 1.29)	0.99 (0.85, 1.15)
House size # of rooms	0.92 (0.76, 1.10)	1.04 (0.87, 1.25)	0.94 (0.74, 1.20)	0.80 (0.55, 1.15)
Wife’s income activity	0.53** (0.28, 0.95)	0.65 (0.34, 1.24)	0.63 (0.29, 1.33)	0.24** (0.07, 0.83)
Food insecurity Yes	1.41 (0.64, 2.95)	0.99 (0.40, 2.45)	1.56 (0.61, 3.96)	0.90 (0.20, 4.03)
Caste: (ref Scheduled caste)				
Scheduled Tribe	0.72 (0.27, 2.03)	2.73* (0.84, 8.91)	1.17 (0.33, 4.17)	0.98 (0.11, 8.46)
OBC	0.60 (0.21, 1.81)	3.09* (0.86, 11.15)	0.56 (0.14, 2.26)	2.28 (0.24, 21.66)
Other	0.39 (0.12, 1.29)	3.55* (0.91, 13.82)	0.50 (0.11, 2.39)	1.88 (0.19, 18.66)

*p < 0.1; **p < 0.05; ***p < 0.001, all estimates are adjusted for all other variables in the model

Model 2 analyses confirmed the importance of contraceptive communication on reported use. Couples in which both report contraceptive communication (*concordant-yes*), relative to those in which both report no communication (*concordant-no*), were more likely to report contraceptive use overall, including *concordant-yes* reports, *Husband + discordant* reports, and *Wife + discordant* reports, relative to couples in which both report non-use of contraception (*concordant-no*). Couples in which men report contraceptive communication and their wives do not (*Husband + discordant*) are also more likely to be those in which both report contraception use (*concordant-yes*), or men only report contraception use (*Husband + discordant*) relative to those in which both report non-use of contraception (*concordant-no*). Couples in which women report communication and their husbands do not (*Wife + discordant*), relative to those in which both report no communication (*concordant-no*), were more likely to be concordant in reporting contraceptive use (*concordant-yes*) or to be *Wife + discordant* relative to couples in which both report non-use of contraception (*concordant-no*). Ideal family size was also related to reported contraception use; couples in which when women prefer more children than husbands (*Wife + discordant*) are more likely to be those in those in which women report contraception use and their husbands do not (*Wife + discordant*).

**Fig. 2:**
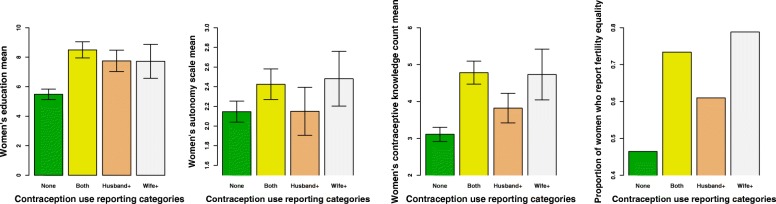
shows the relationship between women’s empowerment and contraceptive use reporting across four different empowerment variables. While women’s education, contraceptive familiarity, and perception of fertility equality is associated with contraceptive use across all three reporting categories, women’s autonomy is associated with concordant and Wife+ discordant reporting

**Fig. 3:**
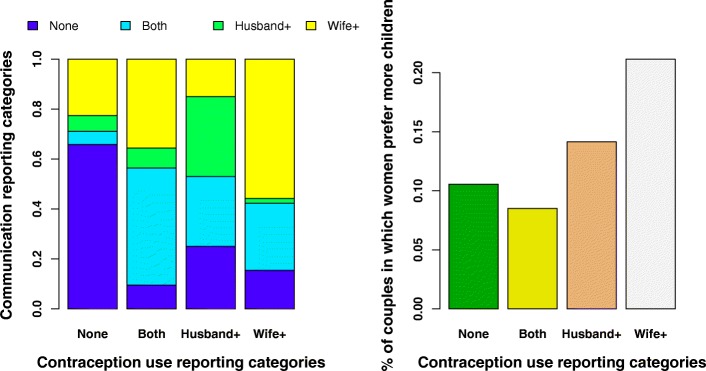
illustrates the distribution of contraceptive communication and family size preferences across contraception use categories. On the left, we see that discrepancies in reporting of contraception communication and contraception use are consistent. Colored bars represent what proportion of each contraception use category is comprised of each contraception communication category. For instance, *Wife + discordance* in contraception use reporting is most strongly associated with *Wife + discordance* in contraceptive communication (the largest yellow bar). On the right we see that over 20% of the Wife+ discordant on contraceptive use couples are those in which women prefer more children than their husbands, the largest proportion across all four categories

In terms of women’s empowerment variables, Model 2 analyses indicate that couples in which women believe they have equality in fertility decision-making are more likely to report contraceptive use overall, either *concordantly* (2.14 times more likely AOR 2.14 95% CI [1.33, 3.44]); *Husband +* discordant 1.78 times more likely (AOR 1.78 95% CI [1.05, 3.01]) or *Wife +* discordant 2.5 times more likely (AOR 2.55 (95% CI [1.17, 5.54]). When we remove contraception communication from the models however, (see Table 6), we find that almost all of the women’s empowerment variables that predicted contraception communication reporting also report contraception use reporting, both *concordantly* and *Wife + discordant*. Figures 2 and 3 illustrate the associations between contraception use reporting categories and communication use categories, ideal family size preferences, and women’s empowerment variables. Again, husband’s education is associated with *Husband + discordant* reporting, as is number of children.

**Table 6:** Odds ratios (95% CI) from a multivariate logistic regression analysis and a multinomial logistic regression analysis looking at the predictors of differences in reported current contraceptive use within couples, rural Maharashtra India, *N* = 843 couples, excluding contraceptive communication

	Multivariate logistic regression	Multinomial multivariate logistic regression		
	Model 1	Model 2		
	Discordant vs concordant	Concordant contraceptive use	Discordant Men+ contraceptive use	Discordant Women+ contraceptive use
Fertility Preferences: (ref concordant)				
Discordant Men+ preferences	0.77 (0.41, 1.41)	0.72 (0.39, 1.34)	0.49* (0.21, 1.11)	1.55 (0.59, 4.07)
Discordant Women+ preferences	1.91** (1.05, 3.42)	0.90 (0.44, 1.83)	1.42 (0.66, 3.04)	3.14** (1.20, 8.26)
Women’s empowerment				
Equality in fertility decision-making	1.58* (0.99, 2.55)	2.34*** (1.42, 3.87)	1.56 (0.89, 2.76)	2.76** (1.15, 6.65)
Wife’s Autonomy	1.03 (0.86, 1.23)	1.23** (1.00, 1.50)	1.04 (0.84, 1.29)	1.16 (0.82, 1.64)
Wife’s Educ in years	1.10** (1.02, 1.19)	1.12*** (1.04, 1.21)	1.14*** (1.04, 1.25)	1.17** (1.02, 1.33)
Wife’s Contraceptive familiarity	1.12* (1.00, 1.25)	1.10 (0.98, 1.23)	1.07 (0.93, 1.24)	1.32*** (1.09, 1.60)
Covariates				
Contraceptive familiarity H # reported	H 0.94 (0.79, 1.11)	1.23** (1.03, 1.46)	1.07 (0.86, 1.33)	0.96 (0.70, 1.32)
Extended Family	0.81 (0.48, 1.37)	0.61* (0.36, 1.05)	0.74 (0.38, 1.41)	0.54 (0.22, 1.32)
Arranged Marriage	2.00** (1.13, 3.69)	0.59* (0.35, 1.01)	1.83 (0.84, 3.99)	1.01 (0.38, 2.67)
Number of Children	1.77*** (1.29, 2.43)	1.83*** (1.33, 2.51)	2.32*** (1.56, 3.44)	1.66* (0.96, 2.88)
Wife’s Age in years	0.89** (0.78, 1.00)	1.02 (0.90, 1.15)	0.81*** (0.69, 0.95)	1.00 (0.82, 1.22)
Husband’s Age in years	0.98 (0.88, 1.09)	1.03 (0.92, 1.15)	0.99 (0.87, 1.12)	1.04 (0.86, 1.27)
Husband’s Educ in years	1.09** (1.01, 1.18)	1.13*** (1.04, 1.22)	1.18*** (1.07, 1.30)	1.00 (0.87, 1.15)
House size # of rooms	0.91 (0.76, 1.08)	1.04 (0.88, 1.23)	0.96 (0.77, 1.19)	0.78 (0.55, 1.09)
Wife’s income activity	0.54** (0.29, 0.94)	0.87 (0.49, 1.53)	0.72 (0.36, 1.42)	0.33* (0.10, 1.09)
Food insecurity Yes	1.21 (0.57, 2.45)	0.89 (0.39, 2.00)	1.28 (0.54, 3.01)	1.03 (0.25, 4.33)
Caste: (ref Scheduled caste)				
Scheduled Tribe	0.80 (0.32, 2.10)	3.38** (1.19, 9.60)	1.26 (0.42, 3.78)	0.97 (0.13, 7.16)
OBC	0.80 (0.30, 2.25)	4.26** (1.36, 13.34)	0.96 (0.29, 3.21)	2.28 (0.28, 18.69)
Other	0.52 (0.17, 1.61)	3.98** (1.19, 13.32)	0.74 (0.19, 2.86)	1.15 (0.14, 9.75)

*p < 0.1; **p < 0.05; ***p < 0.001, all estimates are adjusted for all other variables in the model

## Discussion

In this paper we used a unique approach to understand the predictors of spousal communication on contraception and contraceptive use, by focusing on the dynamics behind discordant versus concordant reporting and the gendered mechanisms associated with them. Consistent with other research [11, 12], in our sample men were more likely to report contraceptive use than were women, in most cases use of condoms. Similarly, women were more likely to report communicating about contraception with their partners. This may be the result of the fact that family planning programs have typically sought out women to target for family planning education. Women therefore may have the onus of initiating conversations on family planning, priming them to be more likely to recall their interactions. We saw little that predicted the differences in ideal family size, however, this may be due to the small actual difference between most couples that did respond discordantly.

Women’s empowerment was significantly associated with both spousal communication on contraceptive use and contraceptive use reporting. Women were more likely to report having discussed contraception with their husbands when they are more educated, report higher autonomy, believe they have equality in decision making, and have greater contraceptive familiarity. While these factors predicted reciprocally reported conversations, they were also predictive of interactions in which women report communication and their husbands did not.

We found that the associations between most of the women’s empowerment variables and contraception use reporting were insignificant, with the exception of equality in fertility decisions. However, when we removed contraception communication reporting from the models, the female empowerment associations were significant (Fig. 3). This suggests there is a strong correlation between female empowerment and contraceptive use, and that this association is intricately linked to contraception communication, which itself is strongly correlated with women’s empowerment. These results point to the need to focus on women’s empowerment as a means to increase communication amongst couples, and thereby also potentially increase use of contraception. Furthermore, while empowered women may be more likely to communicate with their husbands regarding contraception, in many cases, their husbands are not reporting or recalling these interactions. Engaging even empowered women may not be wholly effective without the active engagement of their spouses.

It is also important to note that there were couple-level characteristics that predict *Husband + discordant* reporting: husband’s education, husband’s knowledge of contraception, and number of children. These results show us that not only does neglecting men risk losing information regarding contraceptive events, but that this information is not at random. There are characteristics of couples that predict *Husband + reporting*, such that omitting these husbands from surveys and from intervention work could mean that surveys may not reflect the realities of families with these characteristics, and subsequently intervention programs may not account for their needs. Furthermore these actively reporting husbands may be the most receptive to family planning information and omitting them from programs may limit their effectiveness [32].

One of the strongest results of the analysis was the association of spousal communication on contraceptive use with actual contraceptive use reporting (Fig. 3). If either husband or wife reported communicating on contraception both parties were more likely to report using contraception. Most interestingly, when women only reported contraception communication with a spouse they were also more likely to report using contraception when their husband did not, and visa versa. Discordant communication reporting therefore, was not only predictive of contraceptive use across the couple as a unit, but was predictive of individual specific discordant reported use. Again these results point to the importance of including both men and women in surveys and in reproductive health programs. Differentially working with women risks losing important couple-level information, and misses the potential of couples in which men may communicate with their wives more effectively than their wives communicate with them.

An odd but significant association (Fig. 3) was that women who prefer more children than their husbands are more likely to report using contraception when their husbands do not. While this may seem counter-intuitive, it is possible that desiring more children makes a woman more aware of her contraceptive decisions, and therefore more likely to report on them, particularly if her husband’s reproductive preferences are taking precedence over her own.

There are limitations to this analysis. First, we are looking at cross-sectional associations, not only when considering the determinants of contraceptive use and contraceptive communication, but when considering the differential attributes of the respondents across response categories. Second, without qualitative investigation into the reasoning behind the responses, we can only speculate regarding the sources of them. Third, our sample is specific to tribal areas of rural India, so our results may not be relevant for other populations. Finally, because of small samples in some categories, the confidence intervals on some of the odds ratios are large, suggesting the further investigation within larger populations are warranted to give more precise estimates of these associations. Despite these limitations, these findings show robust results highlighting differences across couples within different categories of reporting on contraceptive events.

## Conclusion

Our results suggest that analyses limited to women only reports could be seriously biased. While interviewing both partners requires more time and money, it may be necessary for survey validity. Dynamics such as the tendency for individuals to report both contraceptive communication *and* contraceptive use when their partner does not should be investigated carefully. Husband-only discordant reports highlight the characteristics of men who may be more receptive to family planning messages than are their wives suggesting that inclusion of men within family planning surveys and programs is important for managing response bias, as well as to engage families in which men may be more pro-active about reproductive health than their wives.

Overall, our findings on discordance demonstrate that discordance matters not only as a measurement issue, but as a reflection of fundamental inequalities that exist within these couples, and potentially thwart their reproductive agency. Inclusion of men in family planning programs offers an important opportunity for couples to communicate their preferences, and potentially negotiate reproductive choices together.

## Abbreviations

CCC: Concordance correlation coefficient
CHARM: Counseling Husbands to Achieve Reproductive Health and Marital Equity Study
K: Cohen’s Kappa
SD: Standard deviation
VIF: Variance inflation factor
